# Long-term outcomes of the Atypical Hemolytic Uremic Syndrome after kidney transplantation treated with eculizumab as first choice

**DOI:** 10.1371/journal.pone.0188155

**Published:** 2017-11-14

**Authors:** Luis Gustavo Modelli de Andrade, Mariana Moraes Contti, Hong Si Nga, Ariane Moyses Bravin, Henrique Mochida Takase, Rosa Marlene Viero, Trycia Nunes da Silva, Kelem De Nardi Chagas, Lilian Monteiro Pereira Palma

**Affiliations:** 1 Department of Internal Medicine, University São Paulo State(UNESP), Botucatu, São Paulo State, Brazil; 2 Department of Internal Medicine, Hospital Estadual de Bauru, Bauru, São Paulo State, Brazil; 3 Department of Internal Medicine, University of São Paulo (USP), São Paulo, São Paulo State, Brazil; 4 Department of Internal Medicine, University of Campinas (UNICAMP), Campinas, São Paulo State, Brazil; University of Toledo, UNITED STATES

## Abstract

**Introduction:**

The treatment of choice for Atypical Hemolytic Uremic Syndrome (aHUS) is the monoclonal antibody eculizumab. The objective of this study was to assess the efficacy and safety of eculizumab in a cohort of kidney transplant patients suffering from aHUS.

**Methods:**

Description of the prospective cohort of all the patients primarily treated with eculizumab after transplantation and divided into the therapeutic (onset of aHUS after transplantation) and prophylactic use (patients with previous diagnosis of aHUS undergoing kidney transplantation).

**Results:**

Seven cases were outlined: five of therapeutic use and two, prophylactic. From the five cases of therapeutic use, there was improvement of the thrombotic microangiopathy in the 48 hours following the start of the drug and no patient experienced relapse during an average follow-up of 21 months in the continuous use of eculizumab (minimum of 6 and maximum of 42 months). One patient died at 6 months, due to *Aspergillus* infection. From the two cases of prophylactic use, one patient experienced relapsed thrombotic microangiopathy after 4 months and another patient remained asymptomatic after 16 months of follow-up, both on chronic treatment.

**Discussion:**

The therapeutic use of eculizumab showed to be effective, with improvement of the microangiopathy parameters and persisting up to the end of the follow-up, without relapses. The additional risk of immunosuppression, leading to opportunistic infections, was well tolerated. The prophylactic use showed to be effective and safe; however, the doses and intervals should be individualized in order to avoid relapsed microangiopathy, especially in patients with factor H mutation.

## Introduction

Atypical hemolytic uremic syndrome (aHUS) is a ultra-rare disease, characterized by a disorder of the alternative complement pathway, leading to hyperactivation and causing microangiopathic hemolytic anemia and altered kidney function[1,2], with an incidence of one to two cases per million inhabitants[3]. There is a well-defined genetic basis for almost two thirds of the cases of aHUS, related to an inactivating mutation of the proteins inhibiting the alternative pathway (H factor, I factor, membrane cofactor protein—MCP or CD46—and thrombomodulin) or a gain-of-function mutation of the pathway activating factors (C3 or B Factor). The formation of anti-H factor IgG antibodies is associated with genetic rearrangement in the proteins related to Factor H (CFHR1). Polymorphisms of risk and variants in these genes determine the penetrance of the disease in mutation carriers[4]

Historically, plasmapheresis/plasma infusion (PP/PI) has been used in the management of aHUS. However, 67% of the adult patients with aHUS treated with PP/PI required dialysis or died after 3 years, with a mortality rate of 8% on the first onset and 11% after 3 years of follow-up[5]. Currently, according to several international treatment guidelines[6,7], the treatment of choice for aHUS is eculizumab infusion, approved in 2011 by FDA, with good efficacy and safety results according to prospective studies[8–10]. The administration of eculizumab is associated with improvement in the kidney function and lower rate of recurrence of thrombotic microangiopathy as compared to traditional plasmapheresis and plasma infusion therapies[1,11]. In the post-kidney transplantation period, aHUS is even more challenging, with increased mortality and high rates of recurrence of the disease, ranging from 60 to 90% in the first year[12,13].

The risk of recurrence of aHUS in the kidney graft is correlated with the type of mutation. The kidney transplantation is highly complex in the patients suffering from aHUS, since 50 to 80% of the patients with aHUS may experience TMA in the kidney graft[13,14], with graft survival of 51% in five years [13–15]. The kidney transplantation recipients are exposed to the risk of MAT by factors directly injuring the endothelium such as immunosuppressive drugs (calcineurin inhibitors and mTOR inhibitors), ischemia-reperfusion injury, rejection and post-transplantation infections[2]. The use of related live donor in the patients with aHUS is contraindicated due to the risk of mutations or polymorphisms that are still undetectable[16]. There are reports of aHUS occurring in previously asymptomatic donors after donation, potentially triggered by the surgical procedure (complement amplifying condition)[17]. The use of eculizumab in kidney transplantation has been chosen as a question of priority, deserving investigation in an initiative of cooperation between patient associations and the global registry of aHUS[18]. Older studies suggest the combination of kidney and liver transplantation, aiming to avoid relapses in patients with certain dysfunctions of complement factors [19]. Eculizumab, a terminal complement inhibitor, is approved for the treatment of aHUS to control the thrombotic microangiopathy (TMA) manifestations and to avoid the relapses of the disease. In the post-kidney transplantation, many studies have already described the same effect [20,21]. However, long-term studies combining eculizumab to immunosuppressive agents used in post-kidney transplantation are lacking[22]. The objective was to assess the long-term outcome of post-transplantation relapsed thrombotic microangiopathy and the incidence of adverse events in a cohort of patients primarily treated with eculizumab for aHUS.

## Case studies and methods

It is a prospective cohort, single-center study developed in Universidade Estadual Paulista (UNESP), Botucatu São Paulo State University. The kidney transplantation service of UNESP is reference for a population of 4,085,510 people, 12 dialysis centers and it performs about 120 kidney transplantations per year.

### Study population

All the patients with a diagnosis of atypical hemolytic uremic syndrome (aHUS) after kidney transplantation that used anti-C5 (eculizumab, Alexion Pharmaceuticals, Cheshire, CT, USA) for prevention of recurrence or treatment of aHUS in the period from February 2013 to December 2016 were assessed. Patients whose diagnosis of aHUS was not confirmed after all the study proofs were made available were excluded. All the cases were in exclusive use of eculizumab as primary treatment for thrombotic microangiopathy. Addictionaly a follow-up protocol was established for monitoring of infections and signs of disease recurrence. The study was approved by the Institutional Review Board (ethics number 64312817.1.0000.5411). None of the transplant donors were from a vulnerable population and all donors or next of kin provided written informed consent that was freely given.

#### Diagnosis of aHUS

The diagnosis of aHUS was established by the founding of thrombotic microangiopathy, defined by the triad: microangiopathic hemolytic anemia (decreased hemoglobin, presence of schitocytes, reticulocytosis, increased LDH, negative direct Coombs test), thrombocytopenia or 25% decrease in the number of platelets and worsening of the kidney function, excluding the use of drugs, infections or other potential secondary causes, as suggested in the literature[1,2,23]. The persistence of the clinical condition of thrombotic microangiopathy, despite the removal of potential secondary causes, motivated the specific treatment with eculizumab. All the cases underwent kidney graft biopsy, which was consistent with thrombotic microangiopathy. The antibody-mediated rejection was excluded by the negative investigation of C4d and absence of specific antidonor antibody. All the patients underwent sampling for assessment of ADAMSTS 13 enzyme activity (disintegrin and metalloproteinase with a thrombospondin type 1 motif), and values above 5% put away severe deficiency, i.e., Thrombotic Thrombocytopenic Purpura. At the time of diagnosis, autoimmune diseases were excluded and the serologies for viral diseases were repeated (Table 1). The full clinical history, the results of the graft biopsy, C4d investigation, antidonor antibody, serologies and genetic analysis are available in supplement (S1 Table).

**Table 1:** Steps for the diagnosis of atypical Hemolytic Uremic Syndrome (aHUS) in the study cohort.

Microangiopathic hemolytic anemia (decreased hemoglobin, presence of schistocytes, reticulocytosis, increased LDH, negative direct Coombs test), thrombocytopenia or 25% decrease in the number of platelets and worsening of the kidney function.	
**Activity of ADAMSTS 13 Enzyme**	> 5% excludes severe deficiency
**Suspension or reduction of the dose of calcineurin inhibitors and/or mTOR inhibitors**	Persistence of the condition of microangiopathy suggests aHUS
**Exclusion of viral infections** **(HIV; HTLV I/II; hepatitis B; hepatitis C; cytomegalovirus; Epstein-Barr)**	
**Exclusion of Bacterial Infections** **(blood culture, urine culture, faeces culture)**	
**Exclusion of autoimmune diseases** **(FAN, antiDNAn, ANCAc, ANCAp, rheumatoid factor)**	
**Histology suggestive of microangiopathy**	
**Exclusion of antibody-mediated rejection**	C4d investigation negative in biopsy and absence of antidonor antibody
**Diagnosis of aHUS**	
**Supplementary study with investigation of mutation (aHUS panel)**	

#### Histological analyses

The biopsy analyses were performed by a pathologist experienced in kidney pathology and transplanted kidney. The C4d investigation in all the biopsies was also assessed by immunofluorescence and immunohistochemistry.

#### Antidonor antibody

The antidonor antibody analyses were performed for the class I (A, B and C) and class II (DP, DQ and DR) loci by Luminex single antigen technique. A pre-transplantation fluorescence intensity (MFI) above 1500 and post-transplantation fluorescence intensity above 300 were considered as a positive result.

#### Genetic analysis

The genetic analyses were performed at CENTOGENE lab (Schillingallee 68,18057 Rostock / Germany) and the methodology is described below. For the Atypical hemolytic uremic syndrome panel, the entire coding region of the ADAMTS13, C3, CD46, CFB, CFH, CFHR1, CFHR2, CFHR3, CFHR5, CFI, DGKE, PIGA, THBD genes including 10 bp of intronic flanking sequences were amplified and sequenced. Raw sequence data analysis, including base calling, demultiplexing, alignment to the hg19 human reference genome (Genome Reference Consortium GRCh37) and variant calling was performed using validated in-house software. All identified variants were evaluated regarding their pathogenicity and causality. All variants except benign or likely benign variants are reported. Analysis does not include copy number variations (CNV) or large deletion/duplications. MLPA (multiplex ligation-dependent probe amplification) analyses were performed using SALSA MLPA probemix P236-A3 provided by MRC-Holland to test for deletions or duplications within or including the CFH, CFHR1, CFHR2, CFHR3, CFHR5 genes.

### Groups

The sample was divided into two groups:

1. **Therapeutic Eculizumab Group**: Patients that presented with thrombotic microangiopathy after the transplantation and had diagnosis confirmation of aHUS according Table 1 and were treated with eculizumab. The dosage used was approved by the FDA, i.e., four weekly doses of 900 mg IV during 4 weeks (induction phase); 1200 mg IV in the fifth week, followed by 1200 mg IV every two weeks (maintenance phase) during an indeterminate time[24]. Each eculizumab 10 mL vial contains 300 mg. The infusions were performed into a peripheral access diluted in an equal volume of saline solution at 0.9%, intravenously, over at least 35 minutes, without premedication.
2. **Prophilatic Eculizumab Group**: Patients whose diagnosis of aHUS was already previously known and who used eculizumab to prevent the relapse of thrombotic microangiopathy. A dose of 900 mg IV was given on the day of surgery before reperfusion and an additional dose of 900 mg IV after 24h. Subsequently, the administration was followed with a 1200 mg dose after one week and for an additional 03 weeks (induction phase), and the 1200 mg dose every 15 days was kept (maintenance phase).

#### Monitoring and response to treatment of the thrombotic microangiopathy

The patients receiving eculizumab were monitored through daily laboratory analysis up to the normalization of the hematological parameters and improvement of the kidney function, defined by the the platelet and LDH levels normalization and improvement the creatinine in two consecutive measurements over the period of four weeks. Then, the monitoring is performed on a weekly basis during the first month, on a biweekly basis up to three months and on a monthly basis over six months. From the sixth month, appointments and exams performed on a quarterly basis or at any time in case of infection or clinical signs of microangiopathy. The hemolytic anemia investigation was performed by using blood count associated with platelet count and schistocyte investigation, LDH and haptoglobin measurement. The analyses of the kidney function were performed by the measurement of the serum creatinine and the proteinuria/creatininuria index.

#### Immunosuppression protocol

All the enrolled patients were transplanted with compatible ABO, negative crossmatch by CDC technique and without antidonor antibodies.

**Kidney transplantation with deceased donor:**
1. Patients with panel reactive antibody (PRA) lower than 30%: induction with thymoglobulin (Thymoglobulin; Genzyme^®^) single dose of 3 mg/kg and followed by the combination of tacrolimus, mycophenolate and prednisone. The target blood level of tacrolimus was 4 to 8 ng/mL. As of 2014, due to a change in the service protocol, the maintenance was performed by using the combination of tacrolimus with everolimus and prednisone, aiming at low blood levels of both drugs: tacrolimus up to 8 ng/mL in the first month and below 5 ng/mL thereafter. The target blood level of everolimus was 3 to 8 ng/mL in all the period after transplantation.
2. Patients with PRA above 30%: induction with thymoglobulin at the dose of 6 mg/kg followed by the combination of tacrolimus with mycophenolate and prednisone. The blood levels of tacrolimus were 8–12 ng/mL in the first month, followed by 4–8 ng/mL thereafter.

**Identical HLA live donor**: no induction therapy was performed. The maintenance was performed with the combination of mycophenolate with prednisone.

#### Prophylaxis against infections

All the cases received prophylaxis with antibiotics up to 15 days after vaccination for meningococcus. The patients were vaccinated once for tetravalent meningococcus (A, C, Y, W135), *Haemophilus influenza* every year and pneumococcus every five years.

All the patients received prophylaxis for *Pneumocystis jiroveci* with cotrimoxazol during six months after the transplantation and were preventively monitored with antigenemia for cytomegalovirus on a weekly basis over a period of 3 months.

#### Clinical variables

All the infectious intercurrences or admissions from the time of transplantation up to the end of the follow-up were recorded. The latest creatinine was also recorded and the kidney function (eGFR) was estimated by using MDRD, besides the parameters for outcome of the thrombotic microangiopathy (hemoglobin, platelets, LDH, haptoglobin, and creatinine) at the following time points: immediately before infusion, 48 hours after infusion, in month 6 and in the last follow-up.

#### Statistical analysis

The results were expressed in mean and standard deviation. We used ANOVA analysis for repeated measures in the therapeutic use at the four time points: before infusion, 48 hours after, 6 months after and at the end of the follow-up. We used the Bonferroni analysis for post-test.

## Results

In the study period, we performed 481 kidney transplants and nine cases (1.9%) evolved with clinical manifestations of TMA. Seven of these patients performed therapeutic use (group 1) and two, prophylactic use (group 2). From the group 1, two patients had the treatment interrupted and were excluded of this analysis because of diagnostic confirmation of antibody-mediated rejection. A total of five cases assessed in group **1** and two cases in group **2** (Table 2).

**Table 2:** Demographic and outcome characteristics of the patients treated with eculizumab after the kidney transplantation in the therapeutic use (group 1) prophylactic use (group 2).

id	Age sex	Underlying Disease	Type of Donor	Panel (%)	Induction	Maintenance	TMA Time after Tx	Outcome	Infections	Follow-up time Latest creatinine/eGFR	Mutation analysis
**Group 1—Therapeutic Use**											
**01**	30 male	Indeterminate	Live (first transplant)	0	No induction	MMP; PDN	4 days	Improvement TMA	No infections	42 m 1.6 mg/dL 49.7 mL/min	Negative
**02**	20 male	Indeterminate	Deceased (third transplant)	73	Thymo 6 mg/kg	FK; MMP; PDN	30 days	Improvement TMA	URTI during follow-up	42 m 0.9 mg/dL 103.7 mL/min	heterozygous variant c.3148A>T (p.Asn1050Tyr). Large deletion in genes CFHR1, CFHR3 Deletion in exon 23 of CFH
**03**	36 female	Indeterminate after childbirth	Deceased (first Tx)	0	Thymo 3 mg/kg	FK; EVE; PDN	2 months	Improvement TMA	No infections	7 m 2.1 mg/dL 27.5 mL/min	Negative
**04**	32 male	GNMP II	Deceased (first Tx)	0	Thymo 3 mg/kg	FK; EVE; PDN	1 month and 20 days	Improvement TMA	2 UTI ESBL+ microorganism	9 m 2.2 mg/dL 33.5 mL/min	Heterozygous disease-associated variant c.1246A>p.Ile416Leu Large deletion in genes CFHR1 and CFHR3
**05**	44 female	Indeterminate	Deceased (first Tx)	67	Thymo 6 mg/kg	FK; MMP/PDN	1 day	Improvement TMA–Evolved with death due to infection 6 months after kidney tx	*Aspergillus* infection	6 m 1.6 mg/dL 34.7 mL/min	Not investigated
**Group 2—Prophylactic Use**											
**06**	29 female	aHUS	Deceased (second Tx)	84	Thymo 6 mg/Kg	FK; MMP; PDN	Preventive use (performed TMA)	Good initial response / graft loss due to arterial thrombosis after 4 months	No infection	4 m 1.2 mg/dL 52.1 mL/min	Heterozygous pathogenic variant c.2056+1G>A
**07**	17 female	aHUS	Deceased (first Tx)	0	Thymo 3 mg/kg	FK; MMP; PDN	Preventive use (performs, performed TMA)	No evidence of TMA	No infection	16 m 0.7 mg/dL 107.8 mL/min	heterozygous variant c.1067G>A (p.Arg356His

FK: tacrolimus; MMP: mycophenolate; EVE: everolimus; PDN: prednisone; THYMO: thymoglobulin; TMA: thrombotic microangiopathy; URTI: upper respiratory tract infection; UTI: urinary tract infection

### Group 1- Therapeutic use of eculizumab in kidney transplantation (n = 5)

Most patients were male with an average age of 32 ± 9 years and received a kidney from decesead donor. In this group, only one patient received a transplant with identical live donor (Table 2). The predominant underlying disease was indeterminate. The panel reactive antibody was 28 ± 38.4%. Most patients (n = 4) received induction therapy with thymoglobulin. The predominant maintenance therapy was tacrolimus, associated with mycophenolate or everolimus, combined with prednisone. The time for onset of thrombotic microangiopathy after the kidney transplantation was 29 ± 26 days (minimum of 1 day and maximum of 60 days). The start of treatment with eculizumab was until 48hs after the diagnostic confirmation of aHUS. The average follow-up time after the start of the treatment was 21.2 ± 19 months (minimum of 6 and maximum of 42 months).

#### Genetic analysis of group 1

Four patients underwent genetic analysis as outlined. Two patients had in H factor and I factor sequence panel alterations, respectively, and for other two cases there were no identifiable mutations and in the fifth patient the mutation was not investigated (Table 2). Patient number 2 had a heterozygous variant c.3148A>T (p.Asn1050Tyr) in the analysis of sequencing of the H factor classified as class 3 (variant of uncertain significance). The patient also has a homozygous large deletion in genes CFHR1, CFHR3 classified as class 1 (pathogenic). Another pathogenic deletion was detected in exon 23 of CFH classified as class 2 (likely patogneic). The Patent number 4 had altered in the analysis of sequencing of the CFI gene (heterozygous disease-associated variant c.1246A>p.Ile416Leu) classified as class 6 (disease associated variant) and in the analysis of MPLA, homozygous large deletion in genes CFHR1 and CFHR3 classified as class 1 (pathogenic).

#### Outcome of thrombotic microangiopathy and kidney function in group 1

**Fig 1:**
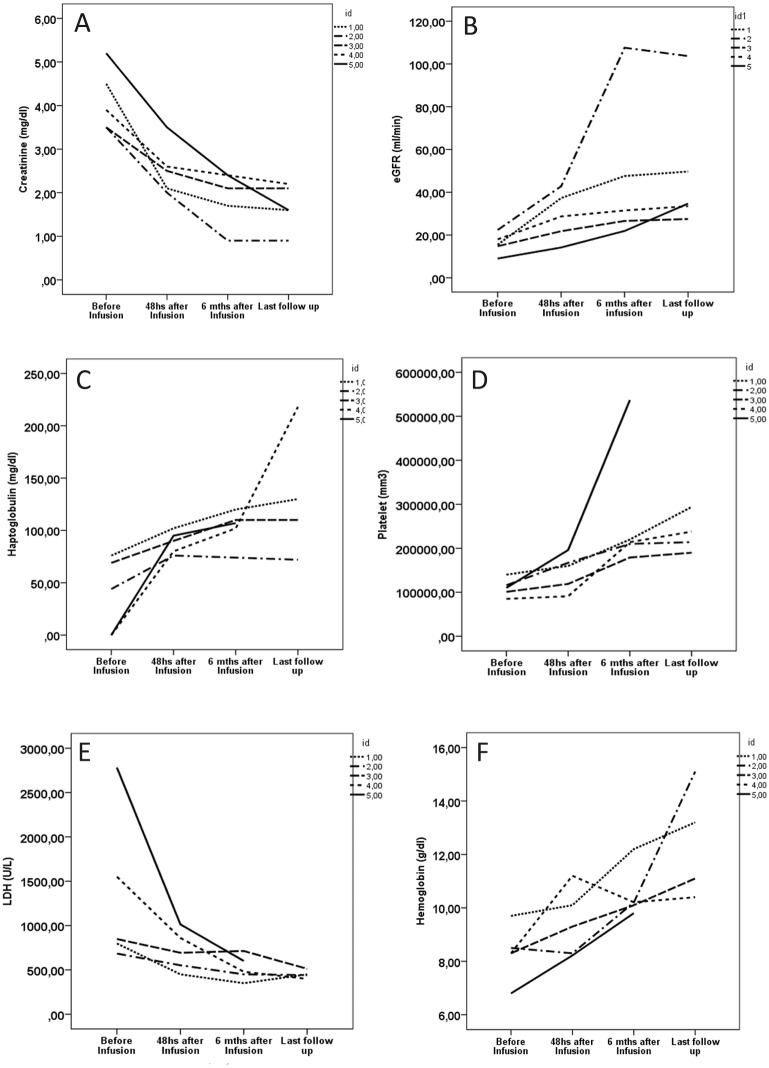
Individual outcome of the hematological and kidney parameters of the patients on therapeutic use (group 1) of eculizumab after the kidney transplantation at time points: Immediately before infusion, 48 hours after, 6 months and at the end of follow up. A: creatinine; B: estimated kidney function (eGFR); C: haptoglobin; D: platelets; E: LDH (lactate dehydrogenase); F: hemoglobin.

All the patients presented clinical response after eculizumab infusion, evidenced by the improvement of the hemoglobin, platelet, LDH and haptoglobin levels after 48hs. It was sustained at six months and at the end of follow-up (Table 3, Fig 1). Haptoglobin and serum creatinine levels presented early clinical response (48 hours after infusion) and no patient experienced relapses of the disease during the follow-up.

**Table 3:** Hematological parameters and kidney function of the patients on therapeutic use of eculizumab after the transplantation at time points: Immediately before infusion, 48 hours after, 6 months and at the end of follow up.

	Mean / Standard Deviation	p
**Hemoglobin (g/dL)**		
Before infusion	8.3 ± 1	
48hs After Infusion	9.4 ± 1.3	
6mth After Infusion	10.5 ± 0.9	0.006
Last follow up	12.4 ± 2.1*	
**Platelets (mm3)**		
Before infusion	110,400 ± 20,255	
48hs After Infusion	146,600 ± 41,500	
6mth After Infusion	272,000 ± 148,984*	0.004
Last follow up	234,000 ± 44,542*	
**LDH (U/L)**		
Before infusion	1332.6 ± 878.1	
48hs After Infusion	713.8 ± 227.1	
6mth After Infusion	518.6 ± 14.1	0.017
Last follow up	450.7 ± 46.5*	
**Haptoglobin (mg/dL)**		
Before infusion	37.8 ± 36.5	
48hs After Infusion	88.6 ± 10.7*	
6mth After Infusion	102.6 ± 17.3*	0.007
Last follow up	132.5 ± 61.8*	
**Creatinine (mg/dL)**		
Before infusion	4.12± 0.73	
48hs After Infusion	2.54± 0.59*	
6mth After Infusion	1.90± 0.63*	0.001
Last follow up	1.68 ± 0.52*	
**eGFR (mL/min)**		
Before infusion	15.9 ± 4.9	
48hs After Infusion	28.9 ± 11.5	
6mth After Infusion	47.1 ± 35.2	0.018
Last follow up	49.8 ± 31.2*	

p: Comparison of the ANOVA repeated measurements between 4 analyzed time points

* p<0.05 x before infusion

All the cases had significant response of the kidney function after the infusion of eculizumab. Pre-infusion serum creatinine was 4.1±0.7 mg/dL, with reduction to 2.5±0.6 mg/dL 48 hours after infusion, 1.9±0.6 mg/dL 6 months after infusion and 1.68±0.52 mg/dL at the end of follow-up, p = 0.001 (Table 3, Fig 1).

#### Survival and infectious intercurrences in group 1

One patient died with functioning graft 6 months after the transplantation due to *Aspergillus* infection (patient number 5). This patient had prolonged hospitalization and use of antibiotics due to infection of the surgical wound and pyelonephritis. Subsequently, *Aspergillus* infection was detected in the surgical wound and in the transplanted kidney, evolving to sepsis and death. Patient number 4 had two episodes of urinary infection by the ESBL+ microorganism motivating the treatment with Ertapenem, with resolution of the condition. The patient number 2 had one episode of viral infection of the upper airways, without repercussions or need for specific treatment.

### Group 2—Prophylatic use of eculizumab in kidney transplantation (n = 2)

All the patients were female with an average age of 23 ± 8.5 years (Table 2). One patient was hypersensitized with PRA of 84% (pre-transplantation) and the other with panel zero. The two cases underwent induction therapy with thymoglobulin and maintenance with tacrolimus, mycophenolate and prednisone. The average follow-up time was 10 ± 8.5 months (ranging from 4 to 16 months).

#### Genetic analysis of group 2

In both cases, presence of mutations in the genetic analysis was identified. The patient number 6 had H factor panel sequence alteration (heterozygous pathogenic variant c.2056+1G>A) classified as class 2 (likely pathogenic) and patient number 7, alterations in the sequencing of gene CFHR5 (heterozygous variant c.1067G>A (p.Arg356His) classified as class 3 (variant of uncertain significance)

#### Outcome of microangiopathy and kidney function in group 2

The hematological parameters of hemoglobin, platelets, LDH and haptoglobin remained normal in the follow-up of patient 7. The patient number 6 had relapse of thrombotic microangiopathy 4 months post-transplantation with graft loss. This patient had a regular follow-up and good renal function after the transplant when she presented hospitalization with vascular complication due to the polongous use of central vein catheters. During hospitalization for correction of central vein stenosis she worsening of progressive renal function accompanied by increased levels of LDL associated with thrombocytopenia. The infusion of additional dose of eculizumab did not reverse the microangipathy and a graft nephrectomy was necessary due to renal artery thrombosis. The patient has been on hemodialysis since then and in regular use of euclizumab.

The serum creatinine at the end of the follow-up was 0.95±0,35 mg/dL and eGFR 79.9±39.4 mL/min, censoring patient number 6 with 4 months after transplantation.

#### Survival and infectious intercurrences in group 2

There were no infectious intercurrences during the prophylactic use in these two cases.

## Discussion

In this study, we reported the outcome of a cohort of patients diagnosed with aHUS primarily treated with eculizumab in the post-transplant period and periodically monitored, aiming to detect relapse of thrombotic microangiopathy. The therapeutical aphaeresis was not performed in these cases because the primary diagnosis was aHUS. According to the literature review, this is the first cohort study in the post-kidney transplantation patients primary treated with eculizumab. From the total series (n = 7), 1 case experienced relapsed thrombotic microangiopathy and 1 case evolved to death due to infection during the chronic treatment with eculizumab considered unrelated to the treatment.

Some issues should be considered in this case series: the first one is the diagnosis of aHUS. As in the post-kidney transplantation, multiple factors may be considered secondary causes of aHUS[12,20], and we separated all the confirmed cases of kidney biopsy and in which we were able to exclude the main secondary causes, such as antibody-mediated rejection, infection or use of calcineurin inhibitors[12]. Only in the cases where the secondary causes were excluded and in the persistence of thrombotic microangiopathy, with histological evidence, the diagnosis of aHUS was confirmed. The high positivity in the genetic analysis investigation supports this diagnosis in this population. Even in the cases where the genetic test was negative, the confirmation could be concluded by exclusion, and it is reported that the genetic test may be negative in 40–50% of the cases[5,25–26].

The second issue is the effectiveness of eculizumab in the treatment of post-kidney transplantation aHUS. The literature reports a good clinical response, but describe cases of therapeutic use associated with the prophylactic use in different dosages and frequency protocols[3,10,20,27–31]. With an administration protocol according to the prescription recommendations[23,24] and as first-line treatment, we showed the efficacy of eculizumab in a short period of time in the therapeutic use (improvement of microangiopathy 48 hours after the start of the therapy) and no patient had relapse of microangiopathy in this group. In the group of prophilatic use one patient had relapse. Similarly, Mallett et al[10], showed in their cohort of 10 cases the absence of response to eculizumab in one patient. Coppo et al reported a child with H factor mutation in whom the use of eculizumab was not sufficient for prevention of relapse of microangiopathy after transplantation[32]. Likewise, other authors [9,33] reported cases that was required increase in the maintenance dose of eculizumab (from 1200 mg to 1500 mg) due to evidence of persistent microangiopathy. In the case of this study, the relapse occurs in a patient with H factor mutation and, therefore, at high risk of relapse; thus, it is hypothesized that there was insufficiency of dose, since it is within the longest interval between applications or because it presents a need for additional boost in the dose. It is established that factors such as infections, trauma, surgery, and pregnancy[1] require additional doses. Then, the clinical condition in this patient that motivating the hospitalization may be a potential factor for activation of the alternative complement pathway.

Several reports of use of eculizumab in the post-transplantation period[3,9,20,27–29] show a short follow-up due to the recent development and availability of the drug that was approved by the US FDA and by the European regulatory agency (EMA) in 2011. In the long-term transplantation studies, we outline the retrospective study by Zuber et al[20] with an average follow-up time of 14.5 months, in which the prophylactic group (n = 9) evolved without relapse and the therapeutic group (n = 13) evolved with disease control. Also in the post-transplantation period, Matar et al[27] reported their retrospective case studies of 12 patients, of which 7 used eculizumab (3 for treatment and 4 as prophylaxis) with satisfactory outcomes in up to 34 months. Since there is no standardization of a protocol for the start of eculizumab in the kidney transplantation, our study showed a good response for treatment of aHUS after transplantation. Differently of the retrospective studies[9,10,20,27] we had a prospective cohort and we used as first choice with a uniform administration protocol. The follow-up time was quite variable, however two cases had follow-up of 42 months without recurrence of microangiopathy. Similar results were found in the extension study of the use of eculizumab in aHUS within up to 2 years[8] and also in the study by Mallett et al with follow-up of up to 19 months[10].

There were two episodes of urinary infection in the same patient of the series, without greater repercussion and upper respiratory tract infection in another patient, also without greater consequence. However, there was one case of severe infection, evolving to death due to *Aspergillus* infection. It is well described that the use of complement alternative pathway blockers may increase the incidence of infections by encapsulated microorganisms, such as *Neisseria meningitidis*, *Streptococcus pneumoniae* and *Haemophilus influenza* type B[11], however there is controversy whether this drug may favor fungal infections[34,35]. On the other hand, this patient underwent prolonged hospitalization, with long use of antibiotics due to infection of the surgical wound, which are also factors of risk for opportunistic infections[36]. This patient also had a long hemodialysis time, prolonged hospitalization and leukopenia, factors associated with the highest incidence of Aspergillus infection in the post-kidney transplantation[37]. Therefore, we can’t surely attribute this episode of infection due to eculizumab use. There were no cases of meningococcal infection or pneumonia during the follow-up period.

Another observation is the results of the genetic analyses. In the second patient of group 1, H factor mutation was evidenced. This patient had a clinical condition of severe microangiopathy in childhood and in the second and third transplants. One patient from group 2, also with H factor mutation, had a severe clinical event and, even with prophylactic use, evolved with graft loss, potentially due to microangiopathy. These data are compatible with highest severity reports in this type of mutation[12,13]. The fourth patient of group 1 had I factor mutation and rearrangements with CHFR1, with the underlying disease defined by membranoproliferative glomerulonephritis. There are reports of association of this glomerulonephritis with aHUS, both caused by complement system derangement[1,38].

The weaknesses of the work reside in the absence of monitoring of the complement system in order to verify the effective blockade of the complement by eculizumab, as suggested by some authors[1,38]. However, the clinical response of the patients was well characterized, as previously described.

The strengths of the work reside in the large number of cases of aHUS, all of them primarily treated with eculizumab, without other interference treatments and, at least in two cases, long follow-up time. The homogeneity in the method of administration of the medication, both in the therapeutic and prophylactic use, cooperates with the relevancy of the data presented. The follow-up of the cases occurred in a prospective manner, with a specific protocol, improving the quality of the information and avoiding the loss of data. The cohorts are an important source of evidence, considering the difficulty in the performance of clinical trials for rare diseases.

## Conclusion

Patients diagnosed with aHUS after kidney transplantation and primarily treated with eculizumab present a very favorable clinical response, with low chance of relapse and the additional risk of immunosuppression is well tolerated.

## Supporting information

S1 Table Detailed description of the clinical history, result of kidney biopsy, autoantibodies, serologies, ADMSTS 13 dose and analyses of mutation of the patients with post-kidney transplantation aHUS treated with eculizumab. * autoantibodies: FAN, antiDNAn, ANCAc, ANCAp; serologies: syphilis, HIV, HTLV I and II; hepatitis B; hepatitis C; Cytomegalovirus; Epstein-Barr
+ Considered Negative class I (A,B,C) and II (DP, DQ and DR) with MFI < 300.
(DOCX)

S1 Dataset Raw data of all the patients included with post-kidney transplantation aHUS treated with eculizumab. The variables are:
Id: identification number; group: 0 = Therapeutic Eculizumab, Group 1 = Prophilatic Eculizumab Group; Age: age in years; sex: 0 = female, 1 = male; Panel: panel reactive antibody; donor: 0 = living, 1 = deceased; Time_MAT: Time MAT in months; Final_creat: Final Creatinin; eGFR_final: final eGFR (ml/min); time_follow_up: time follow up in months; death; graft_loss: graft loss; Creat_before; Creat_48hs; Creat_6months: creatinin before MAT, 48hs after MAT and at 6 months; MDRD_beforer; MDRD_48hs; MDRD_6m: estimated eGFR by MDRD before MAT, 48hs after MAT and at 6 months; platlet_before; platlet_48hs; platlet_6meses; Platelet_final: platlet before MAT; 48hs after MAT; 6 months and at final follow up; DHL_before; DHL_48hs; DHL_6m; DHL_final: LDH before MAT; 48hs after MAT; 6 months after MAT and at final follow up; hapto_before; hapto_48hs; hapto_6m; hapto_final: haptoglobulin before MAT; 48hs after MAT; 6 months after MAT and at final follow-up; Hb_before; Hb_48hs; Hb_6m; Hb_final: hemoglobin before MAT, 48hs after MAT, 6 months after MAT and at final follow-up; T_until_eculizumab: Time until recive eclizumab in months.
(SAV)
