# Supplementary material for: Long-term outcomes of the Atypical Hemolytic Uremic Syndrome after kidney transplantation treated with eculizumab as first choice
Source: PLoS One. 2017 Nov 14;12(11):e0188155. doi: 10.1371/journal.pone.0188155 (PMC5685617; doi:10.1371/journal.pone.0188155)
Supplement: S1 Table — * autoantibodies: FAN, antiDNAn, ANCAc, ANCAp; serologies: syphilis, HIV, HTLV I and II; hepatitis B; hepatitis C; Cytomegalovirus; Epstein-Barr + Considered Negative class I (A,B,C) and II (DP, DQ and DR) with MFI < 300. (DOCX) [file pone.0188155.s001.docx]

**~~Attachment 1. Detailed description of the clinical history, result of kidney biopsy, autoantibodies, serologies, ADMSTS 13 dose and analyses of mutation of the patients with post-kidney transplantation aHUS treated with eculizumab.~~**

| **Id** | **Clinical History** | **Biopsy** | **Autoantibodies^*^**  **New Serologies** | **Post-transplantation antidonor antibody^+^** | **ADMSTS 13**  **(%)** | **Mutation Analysis** |
| --- | --- | --- | --- | --- | --- | --- |
| **01** | Patient underwent kidney transplantation on 08/14/2013 with identical live donor, and initially had good graft function up to the third post-operative. They experienced decrease in creatinine from 11.6 to 2.3 mg/dL. The reactivity panel was negative for class I and II and there was absence of antidonor HLA antibodies. On the fourth post-operative, they experienced increased creatinine and drop in the diuresis volume associated with arterial hypertension. The Doppler ultrasound of the kidneys did not reveal abnormalities. They kept with hypertension and worsened kidney function, with creatinine at 4.5 mg/dL. A kidney biopsy was performed on the sixth post-operative, which was compatible with thrombotic microangiopathy. The patient was not using calcineurin inhibitors as immunosuppression regimen, due to good compatibility with the donor (Identical HLA). Eculizumab infusion was performed, with progressive improvement of the kidney function. | Multiple recent hyaline thrombosis in glomerular capillaries and in one artery. C4d negative | Negative  Negative serologies | Negative | 103  UAH50/mL | Negative |
| **02** | At 02 years-old, the patient experienced a condition of arterial hypertension, anemia, oliguria and acute kidney failure, consistent with Atypical Hemolytic Uremic Syndrome (aHUS), confirmed by kidney biopsy (Kidney Bx 03/11/1999). They needed to start peritoneal dialysis at this time, in which they were kept for 12 months. They underwent kidney transplantation on 04/12/2000 with live donor, and had good graft function and duration of 124 months, with graft loss due to chronic nephropathy. They returned for hemodialysis at this time. The patient underwent a new kidney transplantation with dead donor on 07/02/2011, and had a condition of severe hypertension, seizure and anemia with thrombocytopenia at 100,000 cells/mm³. A kidney Bx was performed, consistent with aHUS (kidney Bx with thrombotic microangiopathy, proliferative endarteritis, and thrombosis). The patient evolved to graft loss, with return to hemodialysis. The patient underwent a new kidney transplantation (third transplantation) with dead donor on 04/06/2013, and evolved again with clinical signs of microangiopathy. Eculizumab was given, with improvement of the microangiopathy and kidney function. | Thrombotic microangiopathy, proliferative endarteritis, and thrombosis.  Cd4 negative | Negative  Negative serologies | Negative | 71 UAH50/mL | sequencing  *CFH* (NM_000186.3): heterozygous variant c.3148A>T (p.Asn1050Tyr)  MPLA  CFHR1, CFHR3: homozygous deletion encompassing the CFHR1 and CFHR3 genes  CFH: homozygous likely pathogenic deletion encompassing exon 23 |
| **03** | The patient had a condition of apathy and weakness, with confirmed end-stage chronic kidney failure of undetermined etiology on 03/25/2013 and start of hemodialysis. The patient reports history of pre-eclampsia during the two pregnancies (2006 and 2009) and need for interruption of the pregnancy before the term. Condition of optical thrombosis in the left eye with sudden vision loss, a week after the first pregnancy in 2006. She had again vision loss of the right eye after the second pregnancy, with ophthalmological diagnosis of optical thrombosis (2009). She keeps only 10% vision of the right eye.  The patient underwent kidney transplantation with dead donor on 01/08/2016, induced with single dose of thymoglobulin (3 mg/kg) and maintenance with tacrolimus, everolimus, and prednisone. The patient had zero panel without antidonor antibodies. The patient evolved with delayed graft function and subsequent recovery of the kidney function, with hospital discharge with creatinine at 4.5 mg/dL and outpatient improvement up to a creatinine at 2.8 mg/dL. At subsequent returns, the patient experienced worsened kidney function and concomitant DHL increase with thrombocytopenia and anemia. A kidney biopsy of the graft was requested, and it was performed 2 months and 7 days following the kidney transplantation, with result suggestive of thrombotic microangiopathy. The patient was shifted from everolimus to mycophenolate, keeping immunosuppression with tacrolimus, mycophenolate and prednisone, and the condition of microangiopathy persisted. Eculizumab infusion was performed, with improvement of the kidney function and microangiopathy. | Hyaline thrombosis in an arteriole next to the glomerular vascular pole. Absence of rejection. Investigation negative for C4d | Negative  Negative serologies | Negative | 80  UAH50/mL | Negative |
| **04** | The patient experienced a clinical condition of arterial hypertension and edema associated with altered kidney function and proteinuria, and they underwent kidney biopsy in November 2014, which revealed membranoproliferative glomerulonephritis. Treatment with corticotherapy 1 mg/kg/day was started without response. The patient evolved with a condition of worsening of the kidney function and hypervolemia, and hemodialysis was indicated in May 2015. The patient underwent kidney transplantation with dead donor on 01/09/2016, induced with single dose of thymoglobulin (3 mg/kg) and maintenance with tacrolimus, everolimus, and prednisone. The patient had zero panel without antidonor antibodies. The patient evolved with delayed graft function and subsequent recovery of the kidney function, with hospital discharge with creatinine at 1.4 mg/dL. During follow-up, the patient remained for 30 days in hospitalization after the transplantation, due to infection of the urinary tract by the ESBL+ microorganism, and was treated during 14 days with meropenem and had a good outcome. The patient experienced a condition suggestive of pyelonephritis with increased CRP and positive urine culture 1 month and 20 days post-transplantation, and they were admitted again and treated with meropenem. At this time, the patient had progressive worsening of the kidney function, despite the established therapy, reaching creatinine = 4.9 mg/dL. One week after the treatment, the patient had, together with the worsened kidney function, increased LDH, anemia with schizocytes and thrombocytopenia, reaching 91.000 cells/mm³ platelets. Hypothesis of thrombotic microangiopathy was performed, confirmed by graft biopsy. Considering the clinical condition, everolimus was replaced with mycophenolate and immunosuppression was maintained with tacrolimus, mycophenolate sodium and prednisone and with persistence of microangiopathy. Eculizumab infusion was performed with improvement of microangiopathy. | Multiple hyaline thromboses predominantly compromising arterioles strongly suggesting  Thrombotic microangiopathy.  Investigation negative for C4d. | Negative  Negative serologies | Negative | 85  UAH50/mL | CFI (NM_000204.3, sequencing): heterozygous disease-associated variant c.1246A>C  (p.Ile416Leu)  CFHR1 and CFHR3 (MLPA, in parallel analysis): heterozygous large deletion encompassing entire  CFHR1 and CFHR3 genes |
| **05** | The patient experiences chronic end-stage kidney failure of indeterminate etiology. At the time, she had history of hypertension, headache and weight loss and confirmed chronic kidney disease, with need for urgency hemodialysis. She underwent kidney transplantation on 01/19/2015 with dead donor. Reactivity panel class I positive 67% and class II negative. Absence of specific antidonor HLA antibodies (MFI < 300). The patient evolved with delayed graft function and normal Doppler ultrasound, discarding surgical complications. She experienced from 01/20/2015 a condition of increased LDG, anemia and thrombocytopenia without evidence of bleeding or perirenal collections (normal abdomen tomography). Immunosuppression with mycophenolate and prednisone. Induction with thymoglobulin (6 mg/kg). Still without use of calcineurin inhibitors (tacrolimus). Assessed by hematology, with presence of several schizocytes per field in a peripheral blood slide, highly suggestive of thrombotic microangiopathy. The patient evolved with need for successive packed red blood cell transfusions as a result of the condition of anemia due to intravascular hemolysis. Graft biopsy with histologically confirmed microangiopathy. Eculizumab infusion was performed, with improvement of the microangiopathy at the final follow-up of up to 6 months. However, the patient evolved with condition of kidney aspergillosis and sepsis, with death 6 months after transplantation. | Extensive kidney parenchymal scarring with glomerular sclerosis (17 sclerotic glomeruli), atrophic tubules,  extensive fibrosis with mild inflammation and hemosiderin deposits. EXTENSIVE SCARRING of the kidney parenchyma suggestive of ISCHEMIC PROCESS.  Negative for C4d. | Negative  Negative serologies | Negative | 61  UAH50/mL | Not investigated |
|  |  |  |  |  |  |  |
| **06** | The patient experienced in May 2006 a condition of anemia and kidney failure triggered after childbirth. At the time, she was admitted to the hospital with a condition of kidney failure and anemia, associated with thrombocytopenia. Atypical hemolytic uremic syndrome or HELLP syndrome was suspected. -(?) The patient evolved with end-stage chronic kidney failure and need for hemodialysis-type replacement kidney therapy.  The patient underwent kidney transplantation on 02/07/2007 with live donor HLA II (haploidentical), and initially had good graft function. The patient evolved with a condition of T-cell-mediated rejection Banff IIB on the eighth post-operative day and underwent pulse therapy with methylprednisolone and good response, with normalization of the kidney function (creatinine = 1.0 mg/dL). On the fifth month after the kidney transplantation, the patient had worsened kidney function with creatinine = 3.8 mg/dL (baseline = 1.0 mg/dL), LDH = 967 U/L (baseline = 520 U/L), haptoglobin = 43 mg/dL (baseline = 249 mg/dL); platelets = 108,000 (baseline = 235,000 cells/mm³) and schizocytes associated with a condition of anemia (Hb=9.7 g/dL) The patient underwent kidney biopsy,: Consistent with thrombotic microangiopathy with marked vascular lesions from proliferative endarteritis with vascular occlusion. Treatment with plasmapheresis and plasma replacement without response, evolving to graft loss. Product of nephrectomy: Consistent with thrombotic microangiopathy with marked occlusion of arterioles and small arteries. Glomerular ischemic atrophy. The patient underwent a new kidney transplantation with dead donor, with infusion of prophylactic eculizumab, since then evolving with good kidney function up to 4 months. At 4 months post-transplantation, the patient needed hospitalization due to the condition of occlusion of superior vena cava and finding of thrombosis of the kidney graft. | Biopsy of the first kidney transplant: Consistent with thrombotic microangiopathy with marked vascular lesions from proliferative endarteritis with vascular occlusion.  Negative for C4d. | Negative  Negative serologies | Negative | 142 UAH50/mL | Sequencing  *CFH* (NM_000186.3): heterozygous likely pathogenic variant c.2056+1G>A |
| **07** | The patient, 17 years old, started urgency hemodialysis on 10/17/2014, with a condition of hypervolemia and significant change of the kidney function. Family history negative for kidney disease. The patient kept thrombocytopenia and anemia, without signs of recovery of the hypertensive kidney function despite treatment with daily hemodialysis, with hypothesis of atypical Hemolytic Uremic Syndrome. Some tests showed hemolysis: Increased LDH 1095 U/L, haptoglobin <8.0 and increased reticulocyte 7.2%. Negative direct Coombs, normal bilirubins, absence of schizocytes in the peripheral blood smear. The patient needed packed red blood cell transfusion, total of 2 units, at 45 days of follow-up. The patient underwent biopsy of the primitive kidney with result suggestive of aHUS. Eculizumab was started, with improvement in the hematological parameters; however, the patient was kept in hemodialysis. The patient underwent kidney transplantation with dead donor on 05/06/2015, with eculizumab prophylaxis, keeping without signs of relapsed microangiopathy and good kidney function. | Biopsy of the primitive kidney:  One fully scarred glomerulus, one glomerulus with mesangiolysis and matrix deposition with mensagial with appearance of collapse. All the other glomeruli showed ischemic collapse. There is still in at least 2 arterioles marked edema and cell proliferation. Negative immunofluorescence. Anatomic-Pathological Diagnosis: consistent with THROMBOTIC MICROANGIOPATHY characterized by endarteritis in 2 arterioles and glomerular ischemic collapse (HEMOLYTIC UREMIC SYNDROME, clinically) | Negative  Negative serologies | Negative | 99  UAH50/mL | Sequencing  *CFHR5* (NM_030787.3): heterozygous variant c.1067G>A (p.Arg356His) |

~~* autoantibodies: FAN, antiDNAn, ANCAc , ANCAp; serologies: syphilis, HIV, HTLV I and II; hepatitis B; hepatitis C; Cytomegalovirus; Epstein-Barr~~

~~+ Considered Negative class I (A,B,C) and II (DP, DQ and DR) with MFI < 300~~
